# Esterification of Levulinic Acid with Different Alcohols Using Mesoporous Stannosilicates As the Catalyst

**DOI:** 10.1021/acsomega.4c04598

**Published:** 2024-07-05

**Authors:** Bruna Ezequielle Bernardes Costa, Antonio Osimar Souza da Silva, Simoni Margareti Plentz Meneghetti

**Affiliations:** † Group of Catalysis and Chemical Reactivity (GCAR), Institute of Chemistry and Biotechnology, Federal University of Alagoas, 57072-970 Maceió, AL, Brazil; ‡ Laboratory of Catalyst Synthesis (LSCAT), Center of Technology, Federal University of Alagoas, 57072-970 Maceió, AL, Brazil

## Abstract

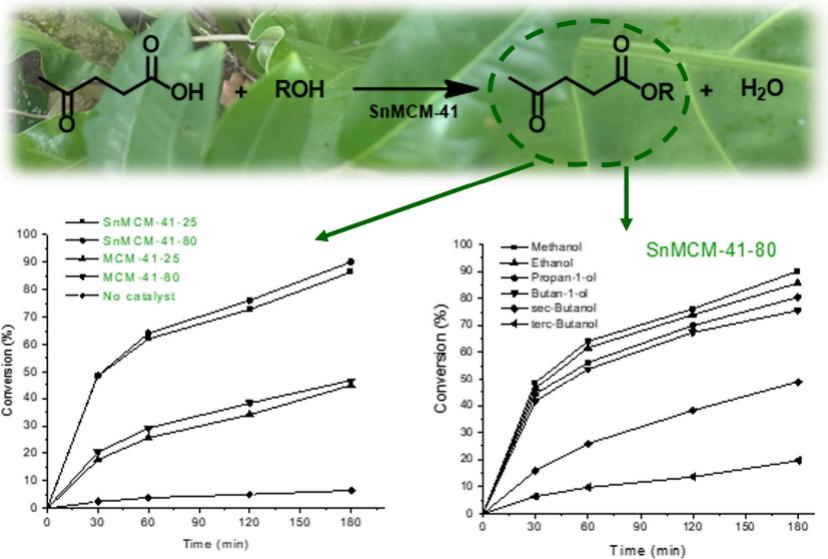

The mesoporous stannosilicates SnMCM-41–25 and SnMCM-41–80, synthesized, respectively, at 25 and 80 °C and exhibiting a well-ordered hexagonal structure, were applied for the first time as heterogeneous catalysts in the esterification of levulinic acid (LA) with different alcohols. The nonhydrothermal method was effective to obtain materials with a high degree of ordering, high acidity, and promising catalytic activity in this esterification. The SnMCM-41–80 led to conversions of 71.0 and 83.6% in 120 and 180 min, respectively, while the respective values for the material without Sn were 33.2 and 40.1% under the same conditions (MeOH:LA molar ratio of 5:1, 1 wt % catalyst, 3 h, 120 °C). In addition, concerning the use of different alcohols, the reaction rate constants (*k*_ap_) were related to the effects of substituents by Taft equation. In general, the polar and steric effects follow the Taft relation, and the length of the chain exerted less influence on the decrease in conversion in comparison to the presence of branches. These results indicate that it is possible to incorporate Sn into the structure of MCM41, thus, making the modified materials more active in the esterification investigated.

## 1 Introduction

The production of chemicals and fuels from alternative and renewable sources is gaining considerable interest in academia and industry with the aim of achieving economic viability through a sustainable process. In this context, lignocellulosic biomass plays an important role as many chemical products can be obtained from it, employing efficient reaction conditions and catalysts for this purpose.^1−3^

Attention has been focused on levulinic acid (LA) and its esters (levulinates) since they can be used for the production of a wide range of molecules that can be applied in the biofuel, polymer, and fine chemicals fields.^4−7^ Alkyl levulinates are valuable compounds used as fuel additives, solvents, and plasticizers, and a notable example is ethyl levulinate (EL), which can be used directly (up to 5 wt %) as a diesel-miscible biofuel since its physicochemical properties are like those of fatty acid methyl esters (FAME).^8−10^

In the reaction to obtain levulinate, mineral acid catalysts, such as HCl and H_2_SO_4_, are generally used. However, they are associated with major disadvantages, for instance, they cannot be recovered and reused, are difficult to handle, and have a high corrosion power, making the process costly and laborious.^11−13^

On the other hand, the use of heterogeneous catalysts is highly desirable, offering the advantage of easy separation of the catalyst (allowing regeneration and reuse), thus, reducing the number of unit operations. Furthermore, the reaction can be carried out in a continuous regime, generating esters with higher purity. There are drawbacks though, since it has been reported that the use of heterogeneous catalysts requires severe reaction conditions and there is a possibility of leaching.^6,8,11^ One auspicious example is the employment of cation exchange resins that leads to promising results and perspective to an industrial application.^14,15^ Another important approach is to apply the immobilized lipase as catalyst to this reaction, since enzymatic esterification becomes a heterogeneous reaction and very good results were attained.^16−18^

The production of levulinates via heterogeneous acid catalysis is thus the focus of research and development aimed at improving the process, and several proposals for the synthesis of solid materials for use as catalysts to obtain levulinates can be found in the literature. In this context, molecular sieves have been highlighted, since they offer the necessary chemical, spatial, and mechanical properties,^19,20^ as well as the possibility of inserting metals into their structure with a view to improving the chemical properties.^5,7,9^ Among the metals incorporated into the silica network present in the structure of mesoporous materials, tin has been shown to be a potential candidate, since with its effective insertion in the lattice, the presence of Brønsted acid sites and Lewis acid sites can be detected.^11,12,21,22^ In the esterification of LA in the presence of ethanol (LA:EtOH in a ratio of 1:3), 50 mg of SnTUD-1 (Si/Sn molar ratio of 100), a material containing interconnected pores, can be used at 4 h and 120 °C, attaining an EL yield of 82.9%.^23^

In this study, the mesoporous stannosilicates MCM-41–25, MCM-41–80, SnMCM-41–25, and SnMCM-41–80 (synthesized, respectively, at 25 and 80 °C) were used, initially, as heterogeneous catalysts in the production of levulinates through the esterification of LA with different alcohols. It is important to note that these materials were obtained for the first time using sodium stannate (Na_2_SnO_3_) as a Sn(IV) source, under mild reaction conditions, and they exhibited a well-ordered hexagonal structure. Therefore, an in-depth investigation of the acidic properties of these materials was carried out to establish a relationship between the acidity and reactivity.

## 2 Results and Discussion

As already reported by our research group, the materials MCM-41–25, MCM-41–80, SnMCM-41–25, and SnMCM-41–80 were synthesized nonhydrothermally at 25 and 80 °C. Sodium stannate (Na_2_SnO_3_) was used as the Sn(IV) source and the amounts of Sn incorporated into the calcined stannosilicates SnMCM-41–25 and SnMCM-41–80 were 2.9% and 3.1%, respectively.^24^ The characterization techniques revealed that silicates and stannosilicates with ordered architecture were obtained under mild reaction conditions and the formation of a mesoporous material with a well-ordered hexagonal structure was verified. The insertion of Sn into the MCM-41 lattice is also confirmed, and the structural characteristics of the support are preserved after modification with tin. These materials have a high surface area and high average pore diameter related to a mesoporous structure and the insertion of tin results in a slight reduction in the specific surface area and an increase in the volume and pore diameter of the support, followed by an increasing in the total acidity.^24^

### 2.1 Acid Properties

**Figure 1:**
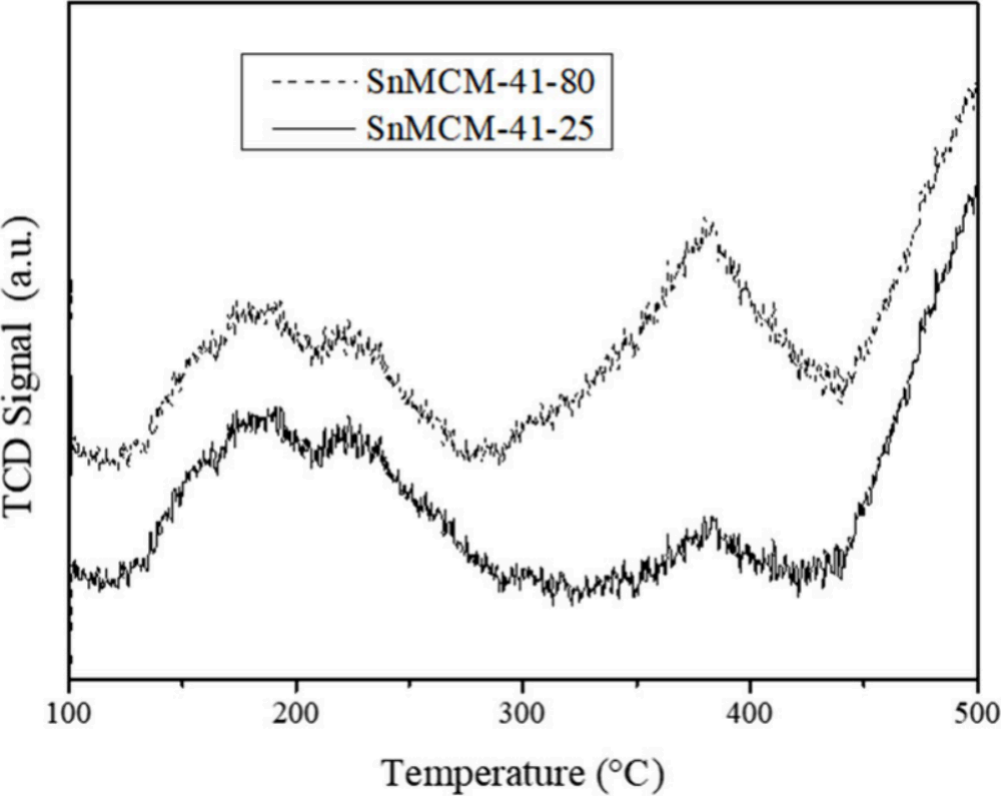
TPD-NH_3_ spectra of SnMCM-41–25 and SnMCM-41–80.

To investigate in-depth the acidic characteristics of these materials, the TPD-NH_3_ profiles were obtained (Figure 1), and the results indicate that the stannosilicates exhibited a desorption peak at low temperature (in the range of 150 to 300 °C), which is attributed to the coordination of NH_3_ to the weak and medium acid sites. It was also possible to observe a second peak centered at approximately 400 °C attributed to the coordination of NH_3_ at strong acid sites, indicating that the insertion of Sn promoted the acidity of the materials since its presence led to a significant increase in the acid strength.

The amount of acid sites, measured considering the desorption peaks, is shown in Table 1. The total amount of moderate acid sites increased with an increase in the synthesis temperature of the materials, while the total amount of strong acid sites decreased with an increase in synthesis temperature. The total acidity was higher for the material synthesized at room temperature.

**Table 1:** Strength and Amount of Acid Sites Obtained by TPD-NH_3_

	acid sites (μmol NH_3_ g^–1^)		
	moderate	strong	total
SnMCM-41–25	9.0	6.0	15.0
SnMCM-41–80	8.9	9.3	18.2

**Figure 2:**
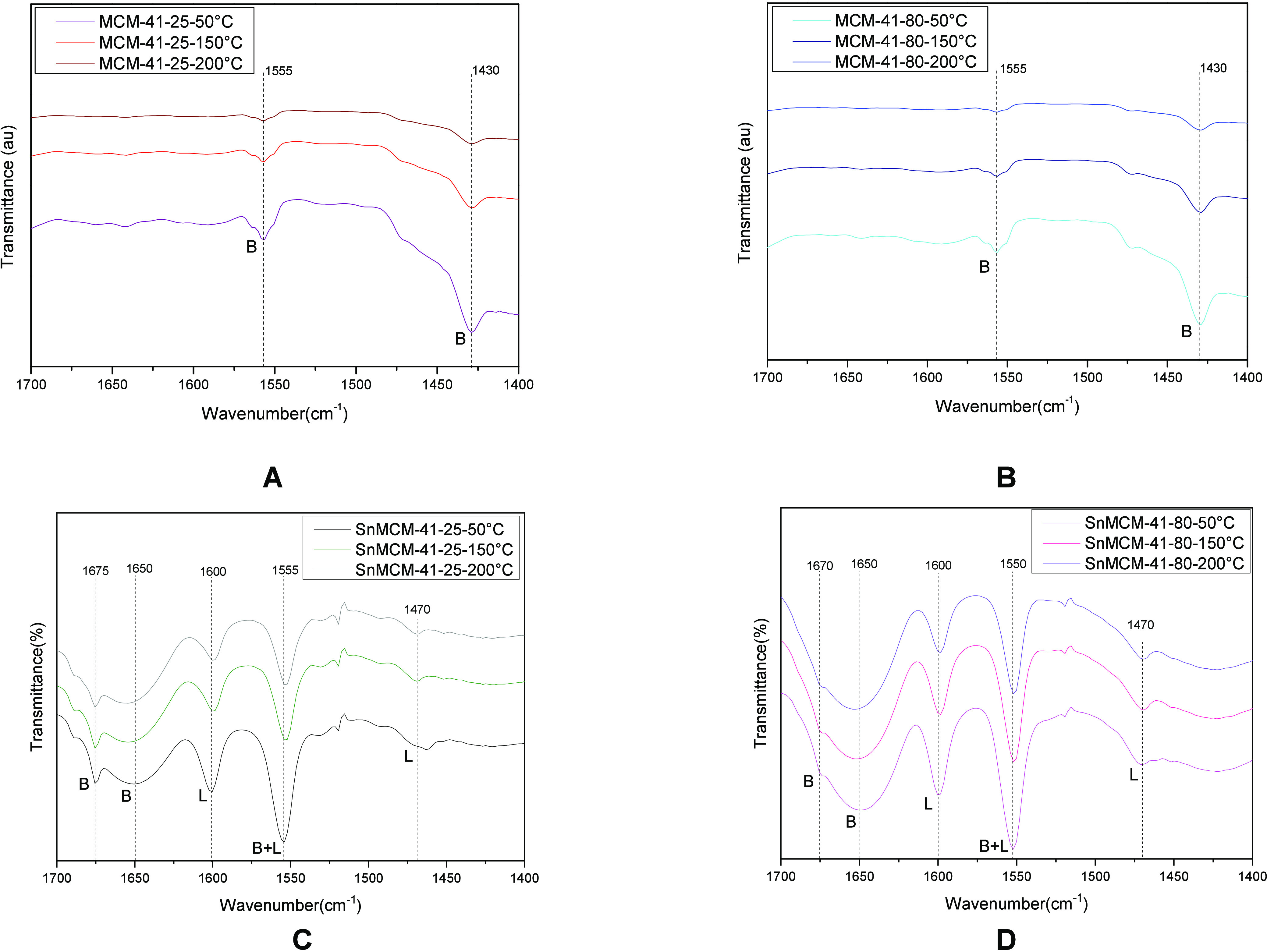
FTIR_py_ of MCM-41–25 (A), MCM-41–80 (B), SnMCM-41–25 (C), and SnMCM-41–80 (D).

The spectrum obtained in the medium infrared region using pyridine as a molecular probe (FTIR_py_, Figure 2) corroborate the TPD-NH_3_ results since the stannosilicates present bands at 1550 cm^–1^ corresponding to Brønsted and Lewis acid sites. The bands at 1600 and 1460 cm^–1^ correspond to strong Lewis acid sites, which are attributed to the incorporation of tin in the tetrahedral coordination of the structure.

According to Figure 2, the bands at 1670 and 1640 cm^–1^ characterize the presence of weak Brønsted acid sites and the band at 1550 cm^–1^ typifies the presence of Brønsted and Lewis acid sites. For the pure materials (MCM-41–25 and MCM-41–80), there are only two bands that correspond to the Brønsted acidity, generated by the interaction between pyridine and the surface silanol groups of MCM-41. As the temperature increases, these bands tend to disappear, since they are fragile interactions, thus, showing that pure materials have only weak Brønsted acidity.

The density of Brønsted and Lewis acid sites present in the catalysts is shown in Table 2, and the results indicate that the increase in temperature promoted an intensification in the density of Lewis and Brønsted acid sites. Furthermore, it is evident that the MCM-41–25 and MCM-41–80 has only Brønsted acid sites in low concentration, and the presence of Sn leads to the formation of Brønsted and Lewis acid sites, promoting a significant increase in their acidic characteristics.

**Table 2:** Specific Surface Area and Amount of Brønsted and Lewis Acid Sites (μmol·g^–1^ and μmol·m^–2^)^a^

sample	*S*_BET_^b^ (m^2^·g^–1^)	*N* Brønsted^c^ (μmol·g^–1^)	*N* Lewis^c^ (μmol·g^–1^)	AS Brønsted^d^ (×10^–2^) (μmol·m^–2^)	AS Lewis^d^ (×10^–2^) (μmol·m^–2^)
MCM-41–25	1156	2.0	nd	0.2	nd
MCM-41–80	1052	2.5	nd	0.2	nd
SnMCM-41–25	846	9.1	7.9	1.1	87.2
SnMCM-41–80	825	10.9	8.9	1.3	81.3

a The amount of acid sites (μmol·g^–1^) was calculated according to eq 1; amount of acid sites considering superficial area (μmol m^2^) was calculated by *N*/*S*_BET_.

b *S*_BET_, superficial area calculated by the BET method.

c *N*, amount of acid sites.

d AS, amount of acid sites considering superficial area.

### 2.2 Catalytic Esterification of LA

**Figure 3:**
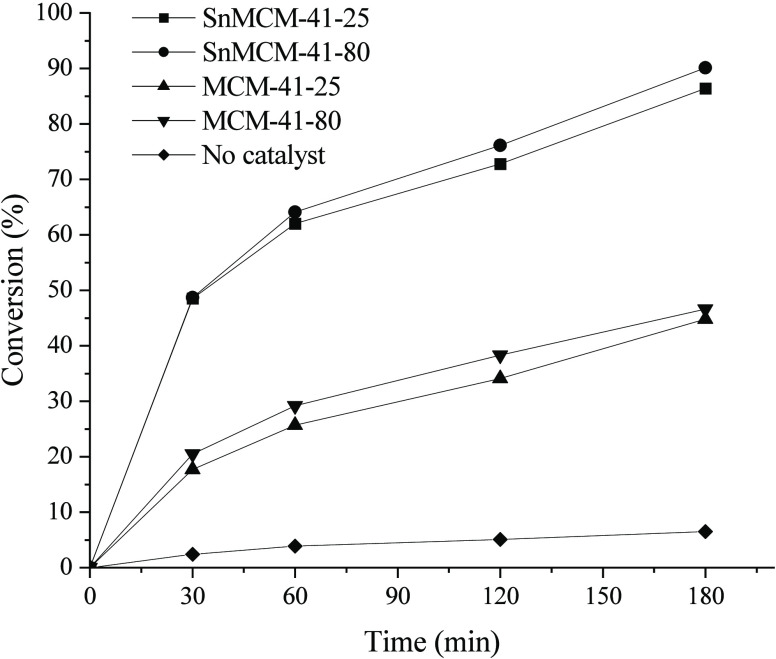
Esterification of LA without catalyst and in the presence of methanol using MCM-41–25, MCM-41–80, SnMCM-41–25, and SnMCM-41–80 (MeOH:LA molar ratio of 5:1, 1 wt % of catalyst, 3 h at 120 °C).

The catalysts were initially tested in the esterification of LA with methanol, with an alcohol/LA molar ratio of 5:1, with 1 wt % of catalyst in relation to the LA weight, applying 3 h of reaction at 120 °C (Figure 3). It is important to note that the esterification of LA is self-catalyzed;^25^ thus, the reaction without catalyst was evaluated under the conditions studied.

The conversion results show that the Sn insertion into MCM-41 enhanced the catalytic activity, corroborating the results obtained by FTIR_py_ spectroscopy and TPD-NH_3_ (Figures 1 and 2). The use of the SnMCM-41–80 catalyst led to the best conversion rates (76.1 and 90.1% after 120 and 180 min, respectively, Figure 3) while the material without Sn provided only 38.3 and 46.6% conversion, respectively, under the same conditions (Figure 3). It is known that to be efficient, the esterification of LA requires the presence of a catalyst with Lewis or Brønsted acidity.^25,26^ Studies suggested that there is a synergistic effect between the Lewis and Brønsted acid sites present in the catalyst structure. Like so, the Lewis sites coordinate with the carbonyl group of LA, while the free −COOH group connects through hydrogen bonds with the substrate, facilitating the departure of the −OH leaving group in LA.^27,28^

In addition, in the case of heterogeneous catalysis the reactions occur mainly on the solid surface, and this confirms the direct dependence on acidity since it is the acidic nature of the surface that determines the course of the reactions.^13,29^

Silica (SiO_4_), which is the constituent responsible for the structural formation of MCM-41, is organized in the form of a network of tetrahedra exhibiting silanol groups (Si-OH) dispersed on the surface, these being the groups responsible for the low Brønsted acidity presented by these materials (Figure 2). It should be noted that, as observed from the FTIR_py_ results, these materials have weak acidity and consequent low reactivity. Thus, the insertion of Sn into the structure of amorphous silica leads to the formation of catalytically active sites in mesoporous molecular sieves.^30,31^

The results obtained show that the materials are promising since they led to high conversion rates. This is mainly due to the increase in acid strength and exposure of the sites since it is a mesoporous material.^32,33^

**Figure 4:**
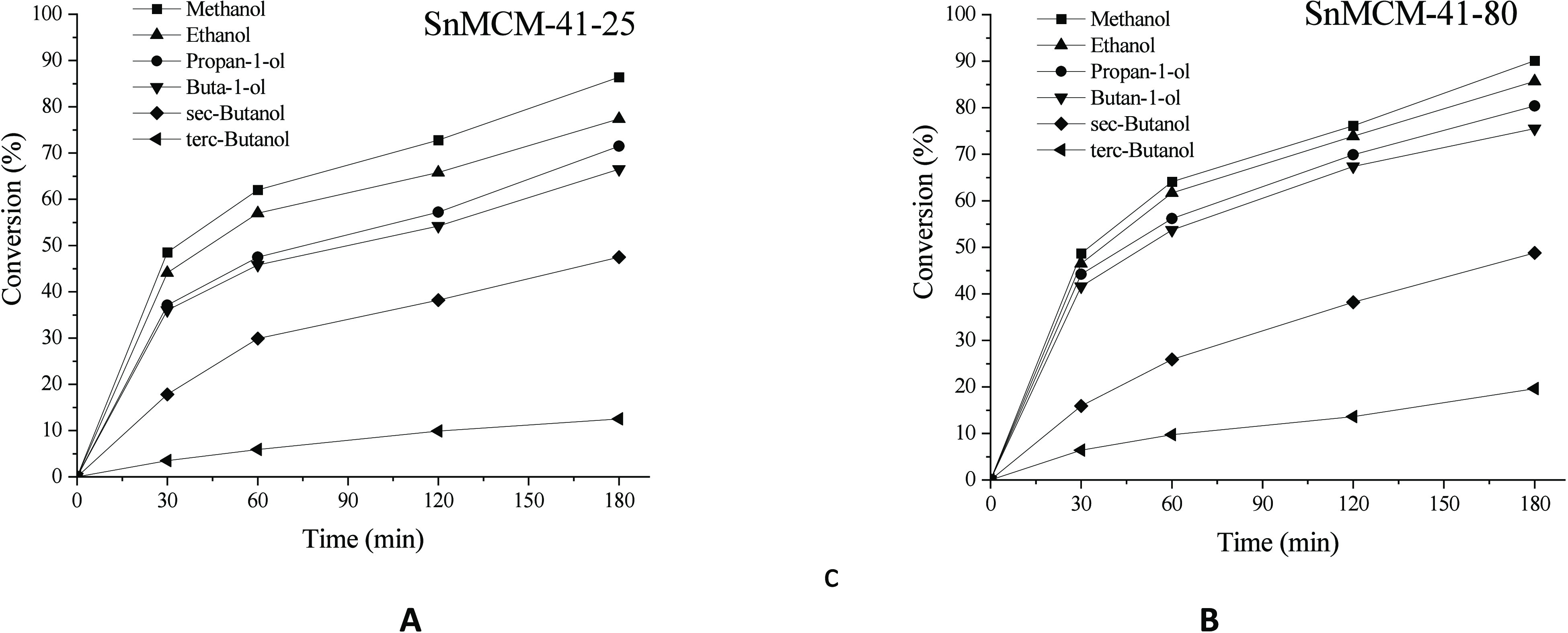
Esterification of LA using MCM-41–25, MCM-41–80, SnMCM-41–25 (A) and SnMCM-41–80 (B), with different alcohols (ROH:LA molar ratio of 5:1, 1 wt % of catalyst, 3 h at 120 °C).

The esterification of LA can be influenced by the nature of the alcohol employed since its characteristics (chain size or steric hindrance close to the hydroxyl group) can hinder the attack of the carbonyl group of the acid, resulting in a lower rate of conversion of the acid to esters. These effects were evaluated using different types of alcohol in addition to methanol: ethanol, propan-1-ol, butan-1-ol, butan-2-ol (sec-butanol), and methyl-2-propanol (*tert*-butanol). The conversion results (Figure 4) show that an increase in the carbonic chain causes a slight decrease in the rate of conversion of LA into esters. In the catalytic tests, it was observed that the alcohol with the shortest chain (methanol) provided a high conversion rate (86.4% for SnMCM-41–25 and 90.1% for SnMCM-41–80). These are very promising results compared to values reported in the literature, since the esters formed have suitable properties for use as gasoline additives.^31−36^

On the other hand, if we evaluate the effect of the presence of branches in the alcohol chain, when secondary and tertiary alcohols were used, the conversion into the respective esters showed a significant drop. This decrease is considerable when compared to, for example, the conversion rate obtained with butyl alcohols using SnMCM-41–25 as the catalyst, with 66.5, 47.5 and 15.9% of conversion being achieved in the reactions with butan-1-ol, sec-butanol and *tert*-butanol, respectively (Figure 4A).

It is important to highlight that in the current study, just as an example, in the presence of SnMCM-41–80 at 120 °C and 3 h, LA conversions of 85.7 and 75.5% were obtained, employing ethanol and n-butanol, respectively. Such results show that the materials are promising since significant conversions were observed, when compared to works in the literature. For example, 50 mg of SnSTUD-1 and LA:EtOH (molar ratio of 1:3) was used for 4 h at 120 °C and an LA conversion ∼83% was achieved.^23^ In the case of LA esterification over aluminum-containing MCM-41 (1%) at 120 °C; using molar ratio of *n*-butanol to LA of 5 and 8 h of reaction, at least 90% of LA conversion was obtained.^37^

**Figure 5:**
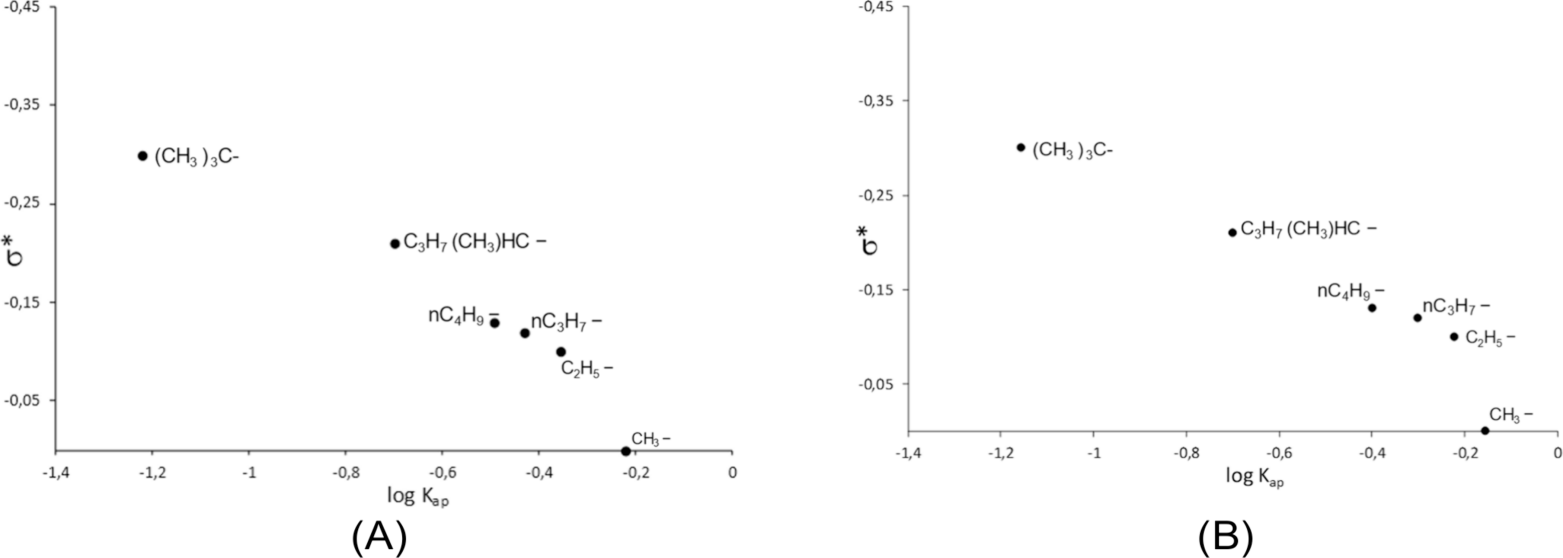
Taft relationship of alkyl radicals in alcohols for SnMCM-41–25 (A) and SnMCM-41–80 (B).

In addition, the rate constants *k*_ap_ (determined as described in the Supporting Information) were related to the polar and steric effects of the substituents (CH_3_^–^, C_2_H_5_^–^, *n*C_3_H_7_^–^, *n*C_4_H_9_^–^, C_3_H_7_(CH_3_)HC^–^, and (CH_3_)_3_C^–^), present at the different alcohols used, according to the Taft eq (Supporting Information), which is applied to aliphatic systems.^38,39^ It is possible to verify that the substituent effects of different alcohols follow the Taft relation for SnMCM-41–25 (*R*^2^ = 0.9724 and 0.9184, without and with CH_3_^–^, respectively) and for SnMCM-41–80 (*R*^2^ = 0.9955 and 0.9033, without and with CH_3_^–^), as illustrated by Figure 5.

In the case of heterogeneous catalysis, it is important to investigate the leaching of the active species into the reaction medium. Therefore, the leaching tests (Figure S1, Supporting Information) indicate that the materials are stable under the reaction conditions evaluated, since, after the removal of the catalysts, the ester conversion ceases, showing that the catalysis phenomenon presented by the materials is truly heterogeneous. Stability, to avoid the leaching of active species into the reaction medium, is one of the main challenges in heterogeneous catalysis, especially in the liquid phase, which favors the release of active species into the reaction medium, leading to the rapid deactivation of the catalyst.^33−35^

**Figure 6:**
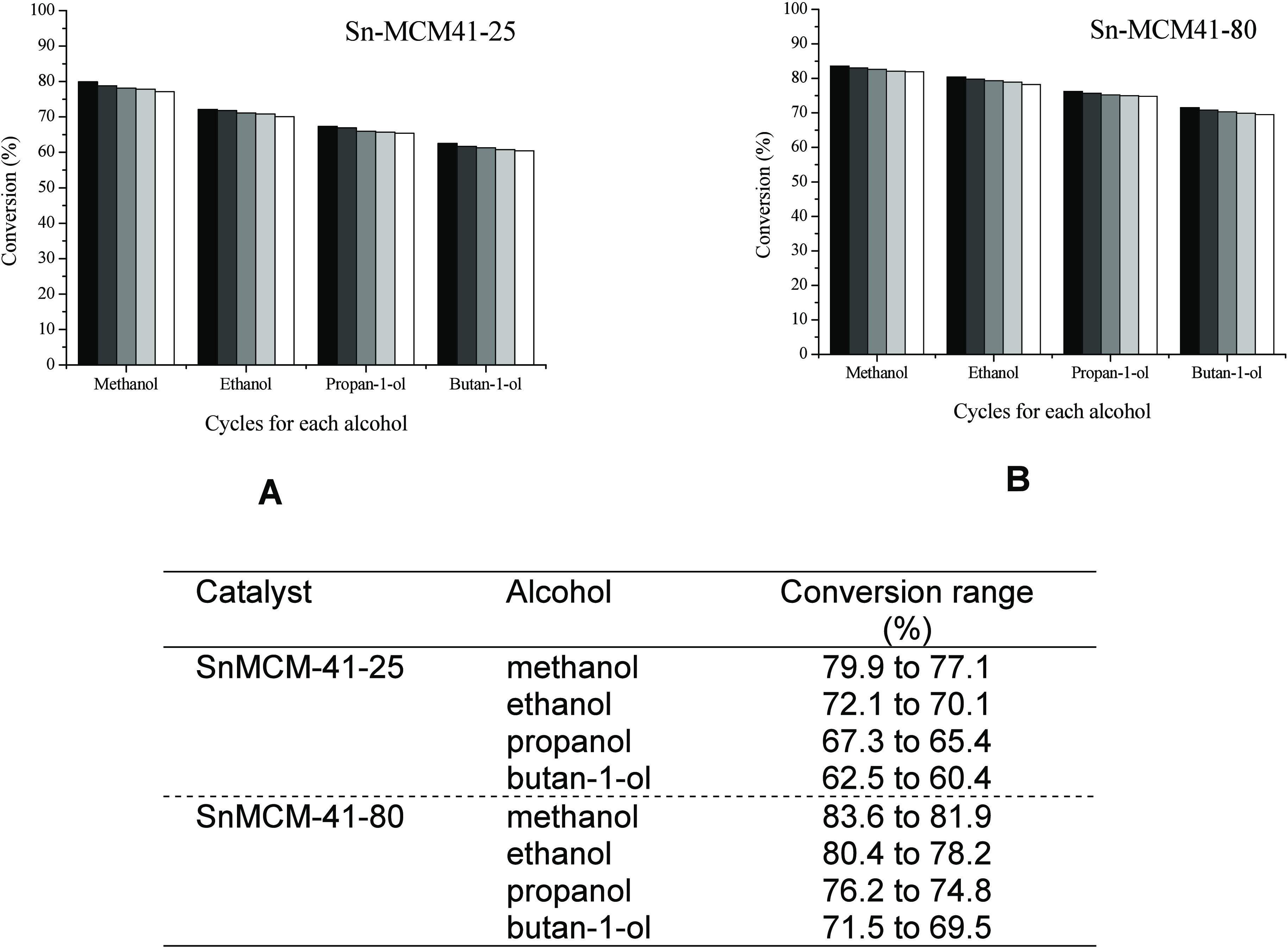
Stability of SnMCM-41–25 (A), SnMCM-41–80 (B) and range of conversion (%).

After verification that there was no leaching of the active species into the reaction medium, the materials were subjected to a reuse study for five consecutive cycles, using different alcohols, to assess their reuse capacity (Figure 6).

It can be observed that the conversion remains practically constant, considering the experimental error, which is around 3.0% (Figure 5). This behavior agrees with the leaching test results shown in Supporting Information, which indicate that the materials are stable and reusable for up to four cycles under the reaction conditions evaluated.

## 3 Conclusions

The synthesis results showed that the replacement of silicon with tin was effective, applying the direct synthesis procedure through the nonhydrothermal method, which means a simple, less laborious, faster, and lower cost method in comparison to those described in the literature. An amorphous material composed of silica with tin incorporated was obtained, exhibiting a hexagonal structure characteristic of type MCM41. The presence of Sn modified the MCM41 structure and significantly increased the acidity of the materials since the density of Brønsted and Lewis acid sites increases for materials containing this metal. MCM-41–25 and MCM-41–80 have only Brønsted acid sites, in a low concentration, and the presence of Sn leads to the formation of Brønsted and Lewis acid sites, promoting a significant increase in the acidic characteristic. The synthesized stannosilicates proved to be active and stable under the reaction conditions evaluated, with high conversions for all primary alcohols used in this study, which allows a range of products that are highly valued to be obtained. In summary, the replacement of silicon by tin in the structure of MCM41, as demonstrated by the synthesis results, opens up new possibilities in the field of material chemistry and catalysis. The nonhydrothermal way used offers an innovative and efficient approach, constituting a direct, simpler, and faster method reducing production time and costs, enabling scalability in more sustainable industrial processes. Even though the synthesized stannosilicates proved to be active and stable under the evaluated characterizations and reaction conditions, studies are underway to determine the catalyst performance degradation and lifetime.

## 4 Experimental Section

### 4.1 Materials

Levulinic acid (LA; 98%), ethanol (98%), and propanol (98.5%) were purchased from Sigma-Aldrich, while 2-methylpropan-2-ol (99.8%), methanol (99.8%), butan-1-ol (99.8%), and butan-2-ol (99.8%) were acquired from Merck. All products were used without further purification.

### 4.2 Catalysts

The synthesis and characterization of the catalysts (MCM-41–25, SnMCM-41–25, MCM-41–80, and SnMCM-41–80) are detailed in a previous publication.^14^ To study the acidic characteristics, temperature-programmed desorption (TPD-NH_3_) measurements were performed using a Termolab multifunctional analytical system (SAMP3). In this analysis, 100 mg of the sample was deposited on quartz wool, and the gas consumption was measured with a thermal conductivity detector (TCD). Brønsted and Lewis acidity measurements were performed by infrared spectroscopy using pyridine as a molecular probe on a Shimadzu IR Prestige 21 spectrometer. Initially, a KBr pellet of the samples was obtained and placed in a container containing liquid pyridine at the bottom without direct contact with the sample. A vacuum was established in the system so that the pyridine was vaporized in the environment. The system remained under these conditions for 24 h until all of the pyridine in the vapor form interacted with the acidic sites of the samples. Spectra were then obtained in the spectral range of 400–4000 cm^–1^. With pyridine adsorbed at the different acid sites, it was possible to determine the amount of acid sites, and monitoring the desorption at 50, 150, and 200 °C allowed the strength of these sites to be determined. The density of Lewis and Brønsted acid sites was calculated using eqs 1 and 2, where *C* = concentration in mmol g^–1^ of Lewis or Brønsted sites; IA(B) = integrated area of the absorption of the Brønsted bands (1675, 1650, 1555, and 1550 cm^–1^); IA(L) = integrated area of the absorption of the Lewis bands (1600 and 1470 cm-1); *R* = radius of the catalyst sample pellet (cm); *M* = mass of the catalyst sample (mg).
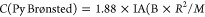
1
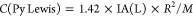
2

### 4.3 Catalytic Evaluation

The catalysts were evaluated in the esterification of LA with different alcohols (methanol, ethanol, propanol, butanol, butan-2-ol, and 2-methylpropan-2-ol). The reactions were carried out in closed glass microreactors with a capacity of 5 mL under magnetic stirring, in batch mode, using the following conditions: alcohol/LA molar ratio of 5:1, with 1 wt % of catalyst in relation to weight of LA, for a reaction time of 3 h at 120 °C. The reaction was monitored by determining the acidity according to the AOCS Cd3D63 method, using eq 3 (*C*(%) = LA conversion; *V*_NaOH_: NaOH volume spent in titration; *C*_NaOH_: NaOH concentration; MM_LA_: molar mass of LA; *M*_s_: sample mass; *f*_c_: relation between LA mass and LA mass + alcohol mass).
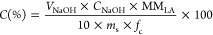
3

The catalyst stability was evaluated by leaching and reuse (deactivation) reactions. To verify the occurrence of leaching of the metal to the reaction medium, a standard reaction was applied, and after 30 min, the catalyst was separated by filtration, and the filtered portion was returned to the reaction. This system was placed under the same reaction conditions for another 2.5 h, and then aliquots were removed to determine the acidity. For reuse of the catalyst after each reaction, the catalyst was washed with the respective alcohol used in the reaction followed by drying at 120 °C for 2 h and reused in 4 more reaction cycles.

## Supporting Information Available

Leaching tests using different alcohols (PDF)
