# Supplementary material for: Esterification of Levulinic Acid with Different Alcohols Using Mesoporous Stannosilicates As the Catalyst
Source: ACS Omega. 2024 Jul 5;9(28):31128–35. doi: 10.1021/acsomega.4c04598 (PMC11256102; doi:10.1021/acsomega.4c04598)
Supplement: Supplementary file 1 — ao4c04598_si_001.pdf [file ao4c04598_si_001.pdf]

Supporting Information

Esterification of levulinic acid with different alcohols using mesoporous stannosilicates as the catalyst

Bruna Ezequielle Bernardes Costa[1,2], Antonio Osimar Souza da Silva[2], Simoni Margareti Plentz Meneghetti[1,]*

[1] Group of Catalysis and Chemical Reactivity (GCAR), Institute of Chemistry and Biotechnology, Federal University of Alagoas, 57072-970 Maceió-AL, Brazil. Fax: 55 82 32141384; Tel: 55 82 32141703; E-mail: simoni.plentz@gmail.com

[2] Laboratory of Catalyst Synthesis (LSCAT), Center of Technology, Federal University of Alagoas, 57072-970 Maceió-AL, Brazil

Figure S1. Leaching tests using different alcohols: **(a)** SnMCM-41-25/methanol; **(b)** SnMCM-41-80/eethanol; **(c)** SnMCM-41-25/ethanol; **(d)** SnMCM-41-80/Ethanol; **(e)** SnMCM-41-25/Propanol; **(f)** SnMCM-41-80/propanol ; **(g)** SnMCM-41-25/butan-1-ol; **(h)** SnMCM-41-80/ butan-1-ol.

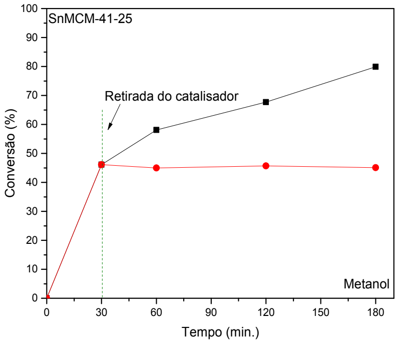

(a)

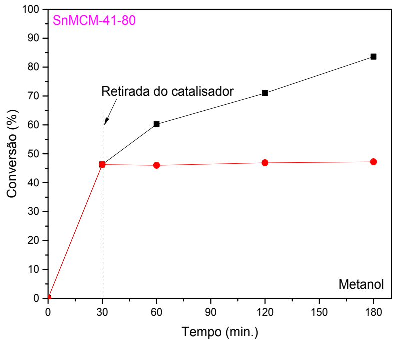

(b)

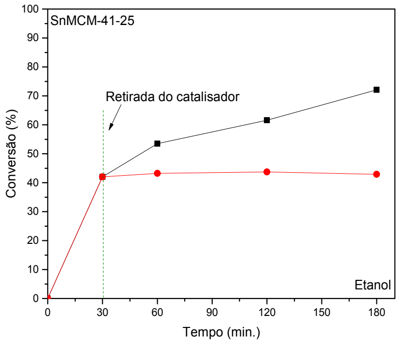

(c)

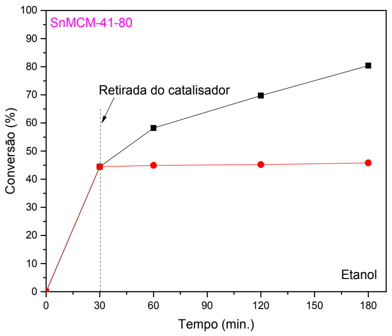

(d)

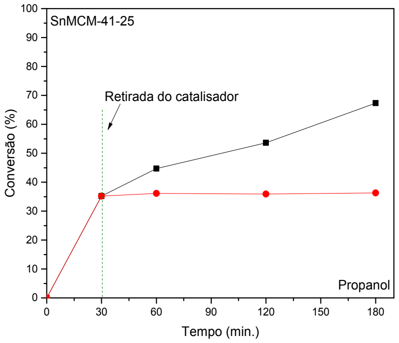

(e)

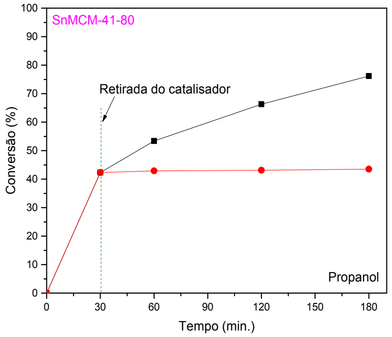

(f)

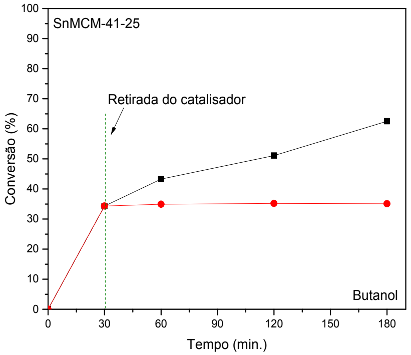

(g)

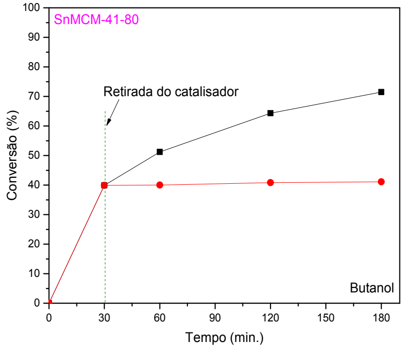

(h)

K_{ap} and Taft relation

Microsoft Excel 2019 software was used for the mathematical treatment of data. The slope of the lines was determined by linear regression, calculating the least squares fit for a straight line represented by Equation S1, where m is the slope and b is the point of intersection.

$$y = mx + b \qquad (S1)$$

Treating the data using the natural logarithm of the LA conversion as a function of reaction time then provides straight lines and their slopes give the values for the apparent rate constants (k_{ap}), according to Equation S2.

$$ln \text{ LA } \% = kt + ln\ 100 \qquad (S2)$$

Taft equation (S3) was used, since is applied to aliphatic compounds, where k = rate constant, k_o = rate constant for the parent compound, ρ^* = polar reaction constant and σ^* is the polar substituent constant.

$$\text{Log } k = \log k_o + \sigma^* \rho^* \qquad (S3)$$

If log (k/k_o) vs σ^* is a straight line (with slope equal to ρ^*), it indicates that the series of the substituted compounds follow the same reaction mechanism and the sum of polar effects and steric effects can be compared.
